# ACTH Stimulation Test for the Diagnosis of Secondary Adrenal Insufficiency: Light and Shadow

**DOI:** 10.3390/biomedicines11030904

**Published:** 2023-03-15

**Authors:** Maria Francesca Birtolo, Simone Antonini, Andrea Saladino, Benedetta Zampetti, Elisabetta Lavezzi, Iacopo Chiodini, Gherardo Mazziotti, Andrea G. A. Lania, Renato Cozzi

**Affiliations:** 1 Department of Biomedical Sciences, Humanitas University, 20072 Pieve Emanuele, Italy; 2 Endocrinology, Diabetology and Andrology Unit, IRCCS Humanitas Research Hospital, 20089 Rozzano, Italy; 3 Fondazione IRCCS Istituto Neurologico Carlo Besta, Unit of Neurosurgery, 20133 Milan, Italy; 4 Endocrinology Department, ASST Grande Ospedale Metropolitano Niguarda, 20162 Milan, Italy; 5 Department of Medical Biotechnology and Translational Medicine, University of Milan, 20122 Milan, Italy

**Keywords:** ACTH Test, adrenal insufficiency, pituitary surgery, new cutoffs

## Abstract

Secondary Adrenal Insufficiency (SAI) is a condition characterized by inappropriately low ACTH secretion due to a disease or injury to the hypothalamus or the pituitary. The evaluation when suspected is often challenging for the non-specific symptoms, the rarity of the disease, and the pitfalls associated with laboratory tests. A prompt and correct diagnosis of SAI is essential because although an adequate hormonal replacement therapy could be lifesaving, inappropriate life-long therapy with steroids can be harmful. The gold standard test for assessing the hypothalamus-pituitary-adrenal axis (HPA) is the insulin tolerance test (ITT), but due to safety issues is not widely used. Conversely, the ACTH stimulation test is a safer and well-tolerated tool for SAI diagnosis. However, data about its diagnostic accuracy show great variability due to both technical and interpretative aspects, such as dose, route of administration, the timing of the test, and assay used for cortisol measurements. Consequently, the clinical background of the patient and the pretest probability of HPA axis impairment become of paramount importance. We aimed to summarize the recent literature evidence in the conduction and interpretation of the ACTH stimulation test for the diagnosis of SAI to provide updated insights on its correct use in clinical practice.

## 1. Introduction

Adrenal insufficiency (AI) can be defined as a defective function of the hypothalamus-pituitary-adrenal (HPA) axis, leading to a non-adequate response of cortisol levels to stress factors. It is a chronic disorder, with an overall prevalence estimated at 300 cases/million people/year [1]. The HPA axis is composed of several components, starting from the secretion of corticotroph-releasing hormone (CRH) in the hypothalamus, which stimulates the transcription of pro-opio-melanocortin (POMC) in the anterior pituitary gland; one of the fragments of POMC is the adrenocorticotrophic hormone (ACTH), which has a direct effect on the adrenal glands, stimulating the secretion of cortisol. This hormone is fundamental for life both being the key element of the stress response in humans and having pleiotropic effects on other organs, such as the action as a counter-regulatory hormone on glycemic homeostasis. Based on its etiology AI can be classified as primary adrenal insufficiency (PAI), due to a reduced function of the adrenal glands, secondary adrenal insufficiency (SAI), caused by impaired production of adrenocorticotrophic hormone (ACTH) in the pituitary gland, and tertiary adrenal insufficiency (TAI), due to prolonged exposure to glucocorticoid (GC) therapy at a supraphysiological dosage which can lead to subsequent persistent suppression of the axis. 

All these patients are at risk of an adrenal crisis, which is a severe and acute episode of AI, characterized by weakness, hypotension, nausea, vomiting, confusion, and typical biochemical alterations (hyponatremia, hyperkalemia, low fasting glucose). It can be life-threatening and requires prompt treatment and urgent hospital care, with its pillar being the administration of intravenous hydrocortisone (HC), and monitoring. From a recent paper published in 2023 and based on Australian records, the admissions for an adrenal crisis are rising in the last two decades, with a stable amount of PAI and an increase in SAI; the authors theorized that the cause of the progressive overcome of adrenal crisis due to SAI rather than PAI are increasing the diagnosis of pituitary adenomas and the use of cancer immunotherapy, which seldom can trigger a hypophysitis [2]. 

An adequate and precocious GC replacement therapy in AI is vital being the key element to preventing an adrenal crisis. Conversely, GC replacement as chronic therapy, in the long run, has also demonstrated detrimental effects, such as an increased incidence of arterial hypertension, type 2 diabetes mellitus, vertebral fractures, reduced bone mineral density (BMD), and increased body mass index (BMI) [3,4].

Considering the dramatic importance but also the risks of GC replacement therapy, the correct diagnosis of AI is pivotal. The diagnosis of PAI is usually overt and is based on clinical signs and symptoms (often the ones of an adrenal crisis), possibly combined with skin hyperpigmentation, very low serum cortisol in the morning, and high ACTH [5] and since confirmatory stimulation tests are not always required, most centers do not routinely perform them. Conversely, in SAI the diagnosis is often challenging. SAI is mainly caused by pituitary diseases, such as macroadenomas and hypophysitis, or therapeutic procedures around the pituitary region, such as trans-nasal-sphenoidal (TNS) surgery and irradiation. The increased rate of diagnosis of pituitary adenomas in the last decades and the minor rate of complications of TNS surgeries compared to open surgeries have increased the amount of patients undergoing these procedures, hence increasing the amount of patients at risk for SAI. Among patients undergoing pituitary surgery in a retrospective study on 519 patients the incidence of new hypopituitarism after 2 months was 3.1%, all requiring hydrocortisone replacement [6]. Oshino et al. demonstrated that the incidence of SAI in macroadenomas correlates with Knosp grade, male sex, and admission for pituitary apoplexy [7]. In addition, cancer therapies, and in particular immunomodulating therapies, became important risk factors in SAI occurrence. In this concern, a recent meta-analysis, among a treated population of 30,014 individuals, 3.2% of the patients had hypophysitis and 0.42% had a following diagnosis of hypopituitarism, with the axes most affected being corticotropic, thyrotrophic, and gonadotrophic [8].

The ACTH stimulation test has been validated against the “gold standard” insulin tolerance test (ITT) to be a reliable tool in the investigation of patients with suspected AI regardless of the underlying cause since it is safer, more convenient, and has no contraindications [9]. The diagnostic value of the ACTH Test in SAI lies in the assumption that chronic ACTH deficiency leads to adrenal atrophy and a consequent impaired response to stimulation by corticotropin analogs [10].

The aim of this paper is to collect and evaluate the most recent available literature on the diagnosis of SAI, with a focus on the role of the ACTH test, in order to provide updated insights on its correct use in clinical practice. We are going to discuss the indication for the test, the patients that should and should not undergo the procedure, the timing to plan it, the preparation of the compound, and how to perform and interpret it. We will also consider the differences between the gold standard test, standard dose ACTH test, and low dose ACTH test in terms of specificity and sensitivity. In conclusion, we will be discussing the new cortisol cut-offs to adopt when the laboratory has a monoclonal method for cortisol determination.

## 2. Methodology

Using the PubMed database, we conducted a literature narrative review on the ACTH Stimulation Test for the diagnosis of SAI to detect the utility and limits of this tool in clinical practice. We identified relevant studies using the following search terms: “ACTH stimulation test”, “cosyntropin stimulation test”, or “short Synacthen Test (SST)” combined with “secondary adrenal insufficiency” or “secondary hypoadrenalism”, “pituitary surgery”, “pituitary disease”, or “trans-nasal-sphenoidal surgery”. We reviewed all the papers on the ACTH stimulation test written in English and then we selected only those on adrenal insufficiency secondary to pituitary surgery or pituitary disease. 

## 3. Patients to Be Tested and Timing for Testing 

According to the Endocrine Society Clinical Practice Guideline for the management of hypopituitarism in adults, in patients undergoing pituitary surgery a morning cortisol sample < 3 μg/dL (82.7 nmol/L) is indicative of AI, while a cortisol level > 15 μg/dL (413 nmol/L) likely excludes an AI diagnosis. Hence, the ACTH stimulation test is suggested in patients with cortisol levels between 3 and 15 μg dL (82.7–413 nmol/L) to exclude an insufficiency of the HPA axis. The guidelines recommend performing the ACTH stimulation test 6 weeks after surgery and at least 18 h after the last HC dose has been administered [11].

Despite data about the timing of postoperative testing are not uniform, with marked variability across centers, from 24 h to 48 h and from 1 to 6 weeks., the most reported timing for testing after pituitary surgery is 6 weeks, while further testing beyond this time point is not regarded as routine and performed in few centers [12].

A recent study demonstrated that the basal cortisol during the ACTH test correlates with the values 30 min after the stimulus and cortisol at baseline ≤ 4.49 μg/dL (124 nmol/L) predicts the failure to reach the peak at the test in 100% of the patients, while a cortisol ≥ 11.38 μg/dL (314 nmol/L) predicts a successful response. In this study, an immunoassay using monoclonal antibodies was used to detect the cortisol levels, and the local laboratory established as a cutoff for the ACTH test failure a cortisol value ≤ 16.3 μg/dL (450 nmol/L) at 30 min after the injection of the corticotropin analog. As discussed in the following paragraph, the interpretation of cortisol value, both at the baseline and stimulated, depends on the assay used for the measurement. According to the results of this study, if the cortisol evaluation is made with immunoassays using monoclonal antibodies, the grey range of 2016 guidelines potentially tighten even more allowing to identify of a narrower population to test [13]. In this context, the most recent studies stressed the importance of selecting patients to test according to the pretest probability of SAI that should be quantified by evaluating the patients’ clinical background and the morning cortisol levels [14,15]. Bioletto et al. proposed an integrated score for the prediction of SAI when morning cortisol is in the grey zone based on morning cortisol levels, sex, and the presence of at least three other pituitary deficits, trying to narrow even more the patients that need to be tested [16].

The morning cortisol level is the most studied predictive factor of SAI, but clinicians should always bear in mind the diurnal variation of cortisol for its correct interpretation and that if the blood test is not performed early in the morning the cortisol value at the baseline has no more a role in the assessment of adrenal function.

## 4. How to Perform the Test 

Several different protocols for ACTH test are reported in the literature with a lack of consensus on the most reliable one, mainly in terms of the dose of corticotropin used, the route of administration, the duration of the test procedure, and the timing of blood sampling. 

According to the standard protocol, cortisol levels should be measured 30 min before and 60 min after intravenous (iv) administration of 250 μg of corticotropin as bolus injection [5]. The low-dose ACTH test is a variation of the standard protocol based on the administration of 1 μg corticotropin for adrenal stimulation. Despite it is still a matter of debate, it seems that the low dose provides the same or even superior diagnostic accuracy compared to the standard one in the evaluation of SAI [9,17,18]. Among these studies, the meta-analysis conducted by Kazlauskaite et al. is relevant since the authors took into account only the published studies on SAI and constructed receiver operator characteristic (ROC) curves using patient-level data from each study and then merged results to create summary ROC curves, adjusting for study size and cortisol assay method. This patient-level meta-analysis found that the diagnostic value of the low-dose ACTH test was superior to the standard-dose test (AUC 0.94 and 0.85, respectively; *p* < 0.001) in the diagnosis of SAI [19]. The same result was found by a more recent study, which concludes that the low-dose ACTH test should be preferred to the 250 μg ACTH test because has a better sensitivity than the standard-dose one since the latter test has more false positives due to supra-physiological adrenal stimulation [18]. In this context, Daidoh et al. found that the minimal dose of ACTH-inducing peak cortisol is 0.5 μg of cosyntropin analog administrated iv, suggesting that even 1 μg leads to supra-physiological adrenal stimulations [20]. 

To perform the ACTH test synthetic corticotropin analogs are used and the two commercially available are supplied as 250 μg/mL ampoules that must be diluted to obtain 1 μg [21]. Therefore, technical remarks arose on the accuracy and reproducibility of making up low-dose tests in terms of dilution methods. In this context, a British survey in 2012 identified 14 different dilution methods of preparing the low-dose test which differed in the amount of the sample utilized for the initial dilution, the volume and the type of diluent, and the number of dilution steps [22]. Based on these findings Cross et al. addressed this crucial clinical issue first identifying through an international survey the ten most used dilution methods and then reporting the accuracy, reproducibility, and reliability of each method. This study showed high variability in both inter-method and intra-method and mostly that the final actual delivered dose of corticotropin analog with the different methods was inadequate in all cases, ranging from 0.16 μg to 0.81 μg, up to seven-fold less than required in some cases. The least variable methods were two: the injection of 1 mL of the 250 μg/mL ampoules into a 500 mL bag of 5% dextrose and the administration of a volume of 2 mL of the resulting solution and the injection of 1 mL of the 250 μg/mL ampoules into 50 mL bag of 0.9% sodium chloride solution (saline), the transfer of 1 mL of this solution to 10 mL syringe containing 9 mL of saline and the administration of a volume of 2 mL. The first one was also the most accurate method with a final real delivered dose close to the desired 1 μg (0.79 to 0.84 μg), but it was the only one to use 5% dextrose as diluent instead of saline and therefore needs to be investigated further before making any recommendation [23]. Being 1 μg an extremely little amount other concerns are about the losses of corticotropin analog when pushed through the devices used for the administration. It was demonstrated that the extent of the loss increases proportionally to the length of the device [24]. A recent study reported that in the low-dose test administrating the corticotropin analog via 2.5-cm plastic tubes or directly intravenous ensures an adequate quantity of corticotropin analog and provides equal cortisol responses [25].

As regards the route of administration most studies were conducted using the IV route, but corticotropin analogs requested for the IV test are not readily available in all countries and although data are limited and mostly obtained in pediatric cohorts the test conducted with intramuscular (IM) long-acting adrenocorticotropic hormone seems to be safe, effective, and reliable in cosintropin the diagnosis of SAI [26,27].

In clinical practice, the ACTH test is performed mostly in the morning [28], but very limited and conflicting data exist on the impact of the time of the day on the test outcome. Previous studies reported that the low-dose test is more prone to abnormal results if conducted in the afternoon [24,29], while more recent analyses showed for the standard dose test no difference in cortisol responses at different times of the day [30]. It is not clear why in the low-dose test the time of the day affects the outcomes test and this aspect should be further investigated in large prospective studies since the interpretation of the ACTH test should be based on the peak stimulated serum cortisol regardless of the basal cortisol value which is influenced by diurnal variation. 

Finally, among the technical aspects, there is still some ongoing debate over the timing of blood sampling after the stimulus. Indeed, since several studies demonstrated that the cortisol peak occurs between 20 and 35 min after the administration of the low dose of corticotropin analog, in clinical practice the cortisol is mostly measured only at baseline and 30 min after the ACTH injection [19]. Nevertheless, the latest evidence suggests that although the majority of patients peak 30 min after the stimulus, measuring cortisol both 30 and 60 min is necessary to reduce the risk of false positives and overdiagnosis of adrenal insufficiency. Indeed, from 10 to 24% of patients, according to several studies, classified as AI based on the 30-min cortisol level are reclassified as adrenally sufficient if the 60-min cortisol level is performed [31,32].

## 5. Alternative Tests for the Assessment of HPA Axis 

Although less often used, other tests to assess the preserved function of the HPA axis and exclude secondary adrenal insufficiency are available. As an example, the glucagon stimulation test (GST) has been described in some papers as an effective option to evaluate this axis. A Russian article published in 2019 used the glucagon stimulation test in 28 patients that underwent craniospinal irradiation for neoplasms of the central nervous system without pituitary localization and in 10 healthy controls, performing both the glucagon and the insulin tolerance test to evaluate whether the HPA axis’ function was preserved or not. A peak of cortisol during the glucagon stimulation test above 18.1 μg/dL (500 nmol/L) ruled out secondary adrenal insufficiency (100% sensitivity), while a peak below 12.2 μg/dL (340 nmol/L) was diagnostic. However, several patients had discordant results (32.2%) between the glucagon stimulation test and insulin tolerance test, and 25% experienced adverse events during the procedure [33]. 

Another more recent study investigated more deeply the safety profile of the test on a wider population, describing 43.2% of patients with adverse effects during the test, mainly characterized by nausea (29.6%), vomiting (27.1%), abdominal cramps (18.5%), but no patient needed to stop the procedure. However, these adverse events were significantly more common in elder patients (*p* = 0.01) [34]. 

A very recent study, published in 2023 by Yalovitsky et al. compared the glucagon stimulation test with the low-dose ACTH test for the diagnosis of SAI in 120 pediatric patients with short stature from Israel. The data collected showed that the glucagon stimulation test had poorer performance than the low-dose ACTH test. The best performance was obtained using the cut-off of 11.18 μg/dL (320 nmol/L), but specificity and sensitivity were respectively 83% and 56% [35]. 

The overnight single-dose metyrapone test has also been used to test patients for the HPA axis function and to rule out secondary adrenal insufficiency. It was very common mainly in the nineties and it consisted of the administration of a single dose administration of 30 mg/kg oral metyrapone and the evaluation of cortisol and 11-deoxycortisol using radioimmunoassay on blood samples obtained the next morning between 08:00 and 09:30 a.m. [36]. This test is different from the stimulation tests (ITT, ACTH test, and GST) since it works by inducing a negative feedback stimulus instead of direct stimulation of the hypothalamus and/or pituitary. Some authors suggest its use in doubt cases. Indeed, overnight single-dose metyrapone was found to be more sensitive than the ITT and standard dose stimulation test in detecting subtle degrees of HPA axis insufficiency [37]. This test has the advantage of not requiring parenteral administration of any drug, but data on its diagnostic accuracy are controversial, some side effects, such as nausea, vomiting, and dizziness, have also been reported and finally the need to have 11-deoxycortisol assays available, has progressively reduced its application [38].

At the moment, no clear data can support the routinary use of the glucagon stimulation test or metyrapone test in place of the ITT and/or the ACTH stimulation test. No comparative studies are available to compare the performance of the glucagon stimulation test to the ACTH test in adults, while data on pediatric populations, although limited to few studies and small cohorts, do not suggest preferring it.

## 6. How to Interpret the Results: Old and New Cortisol Cutoffs 

According to the current guidelines for the diagnosis and management of both PAI and SAI, a peak cortisol level > 18.1 μg/dL (500 nmol/L) at 30 or 60 min after corticotropin analogs administration indicates an appropriate cortisol secretion and therefore excludes the diagnosis of AI [5,11]. This current cortisol cutoff threshold for the diagnosis of AI is based on the old immunological methods that are no longer in use in clinical practice. Indeed, traditionally cortisol cutoff levels across the ACTH test were established with immunoassays using polyclonal antibodies (Elecsys Cortisol generation I) [21], but these assays were characterized by low specificity because they had cross-reactivity with other serum steroids [39,40]. Over the last years, newer-generation assays with greater specificity for cortisol have been developed and have replaced the old assays in almost all the centers. The new tools available to detect cortisol are the immunoassays utilizing monoclonal antibodies, such as Elecsys Cortisol generation II and Beckman Access Cortisol, and the Liquid chromatography–tandem mass spectrometry (LC-MS/MS), which is a non-antibody structural assay highly specific for cortisol [41,42,43,44]. Serum cortisol concentrations measured with the newer assays when compared to those measured with the older ones are sharply lower by 20 up to 36%, according to different studies and assays [45,46,47]. Based on these findings several studies have been published with the aim of identifying the new ACTH-stimulated cortisol threshold values using new cortisol assays; we have summarized the main results of these studies in Table 1. Most of the studies were conducted using the standard dose ACTH test, in small cohorts and without differentiating the cause underlying the AI. Despite these limits, all the studies found a lower cutoff for the 30-min cortisol post-ACTH stimulus ranging from 12.6 μg/dL to 14.6 μg/dL, using the Elecsys Cortisol generation II assay, and from 13.3 μg/dL to 14.9 μg/dL, using the Beckman Access Cortisol assay. All these studies agree on the importance to use the new cutoffs in the presence of the new assays to accurately diagnose AI and to minimize the risk of lifelong glucocorticoid replacement therapy in patients with well-functioning HPA axis. 

If cortisol values are detected with the new assays also the baseline cortisol cutoffs suggestive of AI should be revisited and validated in wide cohorts. Moreover, indications for the management of patients with discrepancies in the basal cortisol value and the stimulated one should be better addressed. 

## 7. When to Repeat the Test?

There are currently few studies on the appropriate frequency of repeat dynamic testing and the eventual likelihood of recovery of HPA axis function, both in patients undergoing pituitary surgery and in those exposed to supraphysiological doses of GC. Pofi et al. demonstrated that HPA axis recovery can occur as late as 9 to 12 months after TNS demonstrating the need for periodic reassessment of patients who initially failed to respond to the ACTH test [30]. The same group found that a 30-min cortisol level above or below 12.7 μg /dL (350 nmol/L) across the first ACTH Test after TNS best predicts HPA axis recovery, enabling early identification of subgroups more likely to recover and hence patients to be prioritized for retesting [31].

## 8. Conclusions

**Figure 1:**
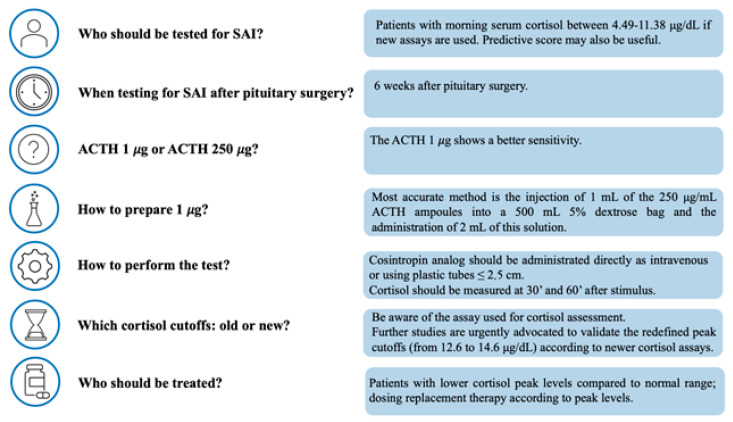
Summary of the latest evidence on the conduction and interpretation of the ACTH test for the diagnosis of SAI. The range of new cutoffs proposed in the figure are from studies conducted using Elecsys Cortisol generation II assay.

A summary of the latest evidence on the conduction and interpretation of the ACTH stimulation test for the diagnosis of SAI is provided in Figure 1. Both standard and low-dose ACTH tests have good diagnostic accuracy in the diagnosis of SAI. The low-dose ACTH test appears to have a better sensitivity than the standard dose one, which probably provides an overstimulation compared to the physiology. Nevertheless, the low-dose test should be performed only in tertiary centers where personnel have the expertise and knowledge of the multiple steps required for the correct preparation and administration. The main issue is the dilution of the 250 μg/mL ampoules, which often leads to a final real delivered dose lower than the desired 1 μg. For the correct interpretation of the results, clinicians must be aware of the assay used for cortisol assessment in their center. If the newer assays are used, the new cortisol cutoffs should be considered for diagnosis, but further studies on both standard and low-dose tests are urgently advocated to validate in a large cohort these redefined cutoffs. Considering the controversies and limits of the test clinicians should select patients to be tested according to the pre-test probability of AI and always correlate the results with clinical findings bearing in mind the dramatic importance but also the risks of the GC replacement therapy.

## Figures and Tables

**Table 1:** Summary of the available studies in the literature on new cortisol cutoffs.

Author, Year	ACTH Dose	Cohort	N. of Patients	30-Min Cortisol Post-ACTH Stimulus			
				Elecsys I	Elecsys II	Access	LC-MS/MS
Raverot et al., 2016 [39]	NA	PAI/SAI	109	18.1 μg/dL (500 nmol/L	13.6 μg/dL (374 nmol/L)	NA	NA
Kline et al., 2017 [48]	250 μg/1 μg	NA	56 *	18.1 μg/dL (500 nmol/L)	12.6 μg/dL (350 nmol/L)	NA	NA
Ueland et al., 2018 [45]	250 μg	PAI/SAI	94	18.1 μg/dL (500 nmol/L)	NA	NA	14.9 μg/dL (412 nmol/L)
Grassi et al., 2020 [47]	250 μg/1 μg	PAI/SAI	30	18.1 μg/dL (500 nmol/L)	12.7 μg/dL (351 nmol/L)	NA	13.3 μg/dL (368 nmol/L)
Javorsky et al., 2021 [44]	250 μg	PAI/SAI/TAI	110	18.1 μg/dL (500 nmol/L)	14.6 μg/dL (402 nmol/L)	14.8 μg/dL (408 nmol/L)	14.5 μg/dL (400 nmol/L)
Zha et al., 2022 [49]	250 μg	NA	50	18.1 μg/dL (500 nmol/L)	NA	13.2 μg/dL (364 nmol/L)	NA
Husni et al., 2022 [50]	250 μg	healthy volunteers	63	18.1 μg/dL (500 nmol/L)	15.7 μg/dL (433 nmol/)	NA	NA

NA: Not applicable; PAI: primary adrenal insufficiency; SAI: secondary adrenal insufficiency; TAI: tertiary adrenal insufficiency; * In the study were included the results of 11-low dose ACTH tests and 45 standard dose ACTH tests, respectively.

## Data Availability

No new data were created.

## Abbreviations

Abbreviations used in the manuscript: PAI (primary adrenal insufficiency), SAI (secondary adrenal insufficiency), TAI (tertiary adrenal insufficiency), CRH (corticotroph-releasing hormone), POMC (pro-opio-melanocortin), ACTH (adrenocorticotrophic hormone), GC (glucocorticoid), TNS (trans-nasal-sphenoidal), ITT (insulin tolerance test), SST (short synacthen test), GST (glucagon stimulation test).

## References

[1] Arlt W., Allolio B. (2003). Adrenal insufficiency. Lancet.

[2] Rushworth R.L., Torpy D.J. (2023). The Changing Epidemiology of Adrenal Insufficiency: Iatrogenic Factors Predominate. J. Endocr. Soc..

[3] Husebye E.S., Pearce S.H., Krone N.P., Kampe O. (2021). Adrenal insufficiency. Lancet.

[4] Falhammar H. (2018). Skeletal fragility induced by overtreatment of adrenal insufficiency. Endocrine.

[5] Bornstein S.R., Allolio B., Arlt W., Barthel A., Don-Wauchope A., Hammer G.D., Husebye E.S., Merke D.P., Murad M.H., Stratakis C.A. (2016). Diagnosis and Treatment of Primary Adrenal Insufficiency: An Endocrine Society Clinical Practice Guideline. J. Clin. Endocrinol. Metab..

[6] Paluzzi A., Fernandez-Miranda J.C., Tonya Stefko S., Challinor S., Snyderman C.H., Gardner P.A. (2014). Endoscopic endonasal approach for pituitary adenomas: A series of 555 patients. Pituitary.

[7] Oshino S., Saitoh Y., Kinoshita M., Mukai K., Otsuki M., Kishima H. (2021). Characteristics of Nonfunctioning Pituitary Adenomas That Cause Secondary Adrenal Insufficiency. World Neurosurg..

[8] Jacques J.P., Valadares L.P., Moura A.C., Oliveira M.R.F., Naves L.A. (2023). Frequency and clinical characteristics of hypophysitis and hypopituitarism in patients undergoing immunotherapy—A systematic review. Front. Endocrinol..

[9] Burgos N., Ghayee H.K., Singh-Ospina N. (2019). Pitfalls in the interpretation of the cosyntropin stimulation test for the diagnosis of adrenal insufficiency. Curr. Opin. Endocrinol. Diabetes Obes..

[10] Garrahy A., Agha A. (2016). How should we interrogate the hypothalamic-pituitary-adrenal axis in patients with suspected hypopituitarism?. BMC Endocr. Disord..

[11] Fleseriu M., Hashim I.A., Karavitaki N., Melmed S., Murad M.H., Salvatori R., Samuels M.H. (2016). Hormonal Replacement in Hypopituitarism in Adults: An Endocrine Society Clinical Practice Guideline. J. Clin. Endocrinol. Metab..

[12] Inder W.J., Hunt P.J. (2002). Glucocorticoid replacement in pituitary surgery: Guidelines for perioperative assessment and management. J. Clin. Endocrinol. Metab..

[13] Ravindran R., Carter J.L., Kumar A., Capatana F., Khan I.N., Adlan M.A., Premawardhana L.D. (2022). Pre-test Cortisol Levels in Predicting Short Synacthen Test Outcome: A Retrospective Analysis. Clin. Med. Insights Endocrinol. Diabetes.

[14] Gasco V., Bima C., Geranzani A., Giannelli J., Marinelli L., Bona C., Cambria V., Berton A.M., Prencipe N., Ghigo E. (2021). Morning Serum Cortisol Level Predicts Central Adrenal Insufficiency Diagnosed by Insulin Tolerance Test. Neuroendocrinology.

[15] Michaelidou M., Yadegarfar G., Morris L., Dolan S., Robinson A., Naseem A., Livingston M., Duff C.J., Trainer P., Fryer A.A. (2021). Recalibration of thinking about adrenocortical function assessment: How the “random” cortisol relates to the short synacthen test results. Cardiovasc. Endocrinol. Metab..

[16] Bioletto F., Berton A.M., Varaldo E., Cuboni D., Bona C., Parasiliti-Caprino M., Prencipe N., Ghigo E., Grottoli S., Maccario M. (2023). Development and internal validation of a predictive score for the diagnosis of central adrenal insufficiency when morning cortisol is in the grey zone. J. Endocrinol. Investig..

[17] Dorin R.I., Qualls C.R., Crapo L.M. (2003). Diagnosis of adrenal insufficiency. Ann. Intern. Med..

[18] Ospina N.S., Al Nofal A., Bancos I., Javed A., Benkhadra K., Kapoor E., Lteif A.N., Natt N., Murad M.H. (2016). ACTH Stimulation Tests for the Diagnosis of Adrenal Insufficiency: Systematic Review and Meta-Analysis. J. Clin. Endocrinol. Metab..

[19] Kazlauskaite R., Evans A.T., Villabona C.V., Abdu T.A., Ambrosi B., Atkinson A.B., Choi C.H., Clayton R.N., Courtney C.H., Gonc E.N. (2008). Corticotropin tests for hypothalamic-pituitary- adrenal insufficiency: A metaanalysis. J. Clin. Endocrinol. Metab..

[20] Daidoh H., Morita H., Mune T., Murayama M., Hanafusa J., Ni H., Shibata H., Yasuda K. (1995). Responses of plasma adrenocortical steroids to low dose ACTH in normal subjects. Clin. Endocrinol..

[21] Grinspoon S.K., Biller B.M. (1994). Clinical review 62: Laboratory assessment of adrenal insufficiency. J. Clin. Endocrinol. Metab..

[22] Elder C.J., Sachdev P., Wright N.P. (2012). The short Synacthen test: A questionnaire survey of current usage. Arch. Dis. Child.

[23] Cross A.S., Helen Kemp E., White A., Walker L., Meredith S., Sachdev P., Krone N.P., Ross R.J., Wright N.P., Elder C.J. (2018). International survey on high and low-dose synacthen test and assessment of accuracy in preparing low-dose synacthen. Clin. Endocrinol..

[24] Wade M., Baid S., Calis K., Raff H., Sinaii N., Nieman L. (2010). Technical details influence the diagnostic accuracy of the 1 microg ACTH stimulation test. Eur. J. Endocrinol..

[25] Saiegh L., Abu-Ahmad A., Sheikh-Ahmad M., Reut M., Chen-Konak L., Jiries N., Shechner C. (2017). Performance of low-dose cosyntropin stimulation test handled via plastic tube. Endocrine.

[26] Sharma R., Madathil S., Maheshwari V., Roy K., Kumar B., Jain V. (2019). Long-acting intramuscular ACTH stimulation test for the diagnosis of secondary adrenal insufficiency in children. J. Pediatr. Endocrinol. Metab..

[27] Ozsu E., Siklar Z., Bilici E., Ceran A., Uyanik R., Cetin T., Aycan Z., Berberoglu M. (2020). Intramuscular Short-term ACTH Test for the Determination of Adrenal Function in Children: Safe, Effective and Reliable. J. Clin. Res. Pediatr. Endocrinol..

[28] Chatha K.K., Middle J.G., Kilpatrick E.S. (2010). National UK audit of the short synacthen test. Ann. Clin. Biochem..

[29] Park Y.J., Park K.S., Kim J.H., Shin C.S., Kim S.Y., Lee H.K. (1999). Reproducibility of the cortisol response to stimulation with the low dose (1 microg) of ACTH. Clin. Endocrinol..

[30] Munro V., Elnenaei M., Doucette S., Clarke D.B., Imran S.A. (2018). The effect of time of day testing and utility of 30 and 60 minute cortisol values in the 250 mcg ACTH stimulation test. Clin. Biochem..

[31] Cartaya J., Misra M. (2015). The low-dose ACTH stimulation test: Is 30 minutes long enough?. Endocr. Pr..

[32] Gill H., Barrowman N., Webster R., Ahmet A. (2019). Evaluating the Low-Dose ACTH Stimulation Test in Children: Ideal Times for Cortisol Measurement. J. Clin. Endocrinol. Metab..

[33] Yudina A.E., Pavlova M.G., Sotnikov V.M., Tselovalnikova T.Y., Mazerkina N.A., Zheludkova O.G., Gerasimov A.N., Teryaeva N.B., Martynova E., Kim E.I. (2019). The glucagon test in diagnosis of secondary adrenal insufficiency after craniospinal irradiation: The feasibility of application, the features of performing the test, and its diagnostic informativity. Probl. Endokrinol..

[34] Ach T., Abdelkrim A.B., Hasni Y., Saad G., Kacem M., Chaieb M., Zaouali M., Maaroufi A., Ach K. (2022). Safety Assessment and Potential Risks of the Glucagon Stimulation Test in the Diagnosis of Secondary Adrenal Insufficiency. Curr. Drug Saf..

[35] Yalovitsky G., Shaki D., Hershkovitz E., Friger M., Haim A. (2023). Comparison of glucagon stimulation test and low dose ACTH test in assessing hypothalamic-pituitary-adrenal (HPA) axis in children. Clin. Endocrinol..

[36] Fiad T.M., Kirby J.M., Cunningham S.K., McKenna T.J. (1994). The overnight single-dose metyrapone test is a simple and reliable index of the hypothalamic-pituitary-adrenal axis. Clin. Endocrinol..

[37] Hartzband P.I., Van Herle A.J., Sorger L., Cope D. (1988). Assessment of hypothalamic-pituitary-adrenal (HPA) axis dysfunction: Comparison of ACTH stimulation, insulin-hypoglycemia and metyrapone. J. Endocrinol. Investig..

[38] Papierska L., Rabijewski M., Migda B., Leszczynska D., Nowak K., Lebek-Szatanska A., Glinicki P., Zgliczynski W. (2022). Evaluation of plasma ACTH in the metyrapone test is insufficient for the diagnosis of secondary adrenal insufficiency. Front. Endocrinol..

[39] Raverot V., Richet C., Morel Y., Raverot G., Borson-Chazot F. (2016). Establishment of revised diagnostic cut-offs for adrenal laboratory investigation using the new Roche Diagnostics Elecsys((R)) Cortisol II assay. Ann. Endocrinol..

[40] Vogeser M., Kratzsch J., Ju Bae Y., Bruegel M., Ceglarek U., Fiers T., Gaudl A., Kurka H., Milczynski C., Prat Knoll C. (2017). Multicenter performance evaluation of a second generation cortisol assay. Clin. Chem. Lab. Med..

[41] Hawley J.M., Owen L.J., Lockhart S.J., Monaghan P.J., Armston A., Chadwick C.A., Wilshaw H., Freire M., Perry L., Keevil B.G. (2016). Serum Cortisol: An Up-To-Date Assessment of Routine Assay Performance. Clin. Chem..

[42] Monaghan P.J., Keevil B.G., Stewart P.M., Trainer P.J. (2014). Case for the wider adoption of mass spectrometry-based adrenal steroid testing, and beyond. J. Clin. Endocrinol. Metab..

[43] Monaghan P.J., Keevil B.G., Trainer P.J. (2013). The use of mass spectrometry to improve the diagnosis and the management of the HPA axis. Rev. Endocr. Metab. Disord..

[44] Javorsky B.R., Raff H., Carroll T.B., Algeciras-Schimnich A., Singh R.J., Colon-Franco J.M., Findling J.W. (2021). New Cutoffs for the Biochemical Diagnosis of Adrenal Insufficiency after ACTH Stimulation using Specific Cortisol Assays. J. Endocr. Soc..

[45] Ueland G.A., Methlie P., Oksnes M., Thordarson H.B., Sagen J., Kellmann R., Mellgren G., Raeder M., Dahlqvist P., Dahl S.R. (2018). The Short Cosyntropin Test Revisited: New Normal Reference Range Using LC-MS/MS. J. Clin. Endocrinol. Metab..

[46] El-Farhan N., Pickett A., Ducroq D., Bailey C., Mitchem K., Morgan N., Armston A., Jones L., Evans C., Rees D.A. (2013). Method-specific serum cortisol responses to the adrenocorticotrophin test: Comparison of gas chromatography-mass spectrometry and five automated immunoassays. Clin. Endocrinol..

[47] Grassi G., Morelli V., Ceriotti F., Polledri E., Fustinoni S., D’Agostino S., Mantovani G., Chiodini I., Arosio M. (2020). Minding the gap between cortisol levels measured with second-generation assays and current diagnostic thresholds for the diagnosis of adrenal insufficiency: A single-center experience. Hormones.

[48] Kline G.A., Buse J., Krause R.D. (2017). Clinical implications for biochemical diagnostic thresholds of adrenal sufficiency using a highly specific cortisol immunoassay. Clin. Biochem..

[49] Zha L., Li J., Krishnan S.M., Brennan M.R., Zhang Y.V., Povse P., Kerlin R., Shively K., Oleksik F., Williams J. (2022). New Diagnostic Cutoffs for Adrenal Insufficiency After Cosyntropin Stimulation Using Abbott Architect Cortisol Immunoassay. Endocr. Pr..

[50] Husni H., Abusamaan M.S., Dinparastisaleh R., Sokoll L., Salvatori R., Hamrahian A.H. (2022). Cortisol values during the standard-dose cosyntropin stimulation test: Personal experience with Elecsys cortisol II assay. Front. Endocrinol..

